# Primary pulmonary meningioma: a case report and literature review

**DOI:** 10.3389/fonc.2025.1601698

**Published:** 2025-07-14

**Authors:** Qinmin Liu, Shuhua Luo, Meijuan Guo

**Affiliations:** ^1^ Department of Radiology, First Affiliated Hospital of Gannan Medical University, Ganzhou, Jiangxi, China; ^2^ Department of Radiology, Ganzhou People’s Hospital, Ganzhou, Jiangxi, China; ^3^ Thyroid and Breast Surgery, Ganzhou People’s Hospital, Ganzhou, Jiangxi, China

**Keywords:** primary pulmonary meningioma, imaging, diagnose, treatment, prognosis

## Abstract

Meningioma is a common tumor of the central nervous system, with occasional occurrences outside this region. Herein, we present a case of primary pulmonary meningioma (PPM) in a 64-year-old male who was both a non-smoking and non-drinking. Computed tomography (CT) imaging unveiled a solitary, lobulated, and well-circumscribed solid nodule in the posterior basal segment of the right lower lobe. This nodule exhibited a mean attenuation of approximately 31.6 Hounsfield units (HU). Upon contrast-enhanced CT, the lesion demonstrated persistent moderate enhancement, with attenuation values rising to 69.7 HU in the arterial phase and further increasing to 78.2 HU in the venous phase. Positron emission tomography-computed tomography (PET-CT) scanning revealed mildly increased fluorodeoxyglucose (FDG) uptake within the lesion, with a maximum standardized uptake value (SUVmax) of approximately 2.4. The patient underwent uniportal video-assisted thoracoscopic surgery (VATS) for wedge resection of the nodule in the right lower lobe. Histopathological examination subsequently confirmed the diagnosis of meningioma, specifically the transitional type classified as WHO grade I. Postoperatively, the patient recovered well, with follow-up chest CT scans demonstrating no evidence of tumor recurrence. PPM is recognized as an extremely rare meningioma of ectopic origin, predominantly benign in nature, and often presenting with asymptomatic clinical manifestations. CT imaging typically shows single or multiple nodules with clearly defined boundaries, uniform density, and smooth edges. Notably, surgical resection has proven to be an effective treatment strategy for PPM.

## Introduction

Meningiomas represent the most prevalent primary central nervous system neoplasms, originating from arachnoid cap cells within the meningeal layers. It can also occur in locations outside the central nervous system, such as skull bones, orbit, nose, paranasal sinuses, neck, skin, lungs, mediastinum and peripheral nerves (1). However, primary pulmonary meningioma (PPM) is extremely rare. The diagnostic evaluation of PPM is frequently complicated by its clinically silent presentation and indeterminate radiological characteristics, which often lead to erroneous classification as either benign pulmonary nodules or malignant neoplasms (2, 3). This diagnostic dilemma presents substantial challenges for both radiologists and clinicians in establishing accurate preoperative diagnoses. Here, we report a case of PPM, providing a complete clinical history, imaging data, surgical records, and pathological results.

## Case report

**Figure 1:**
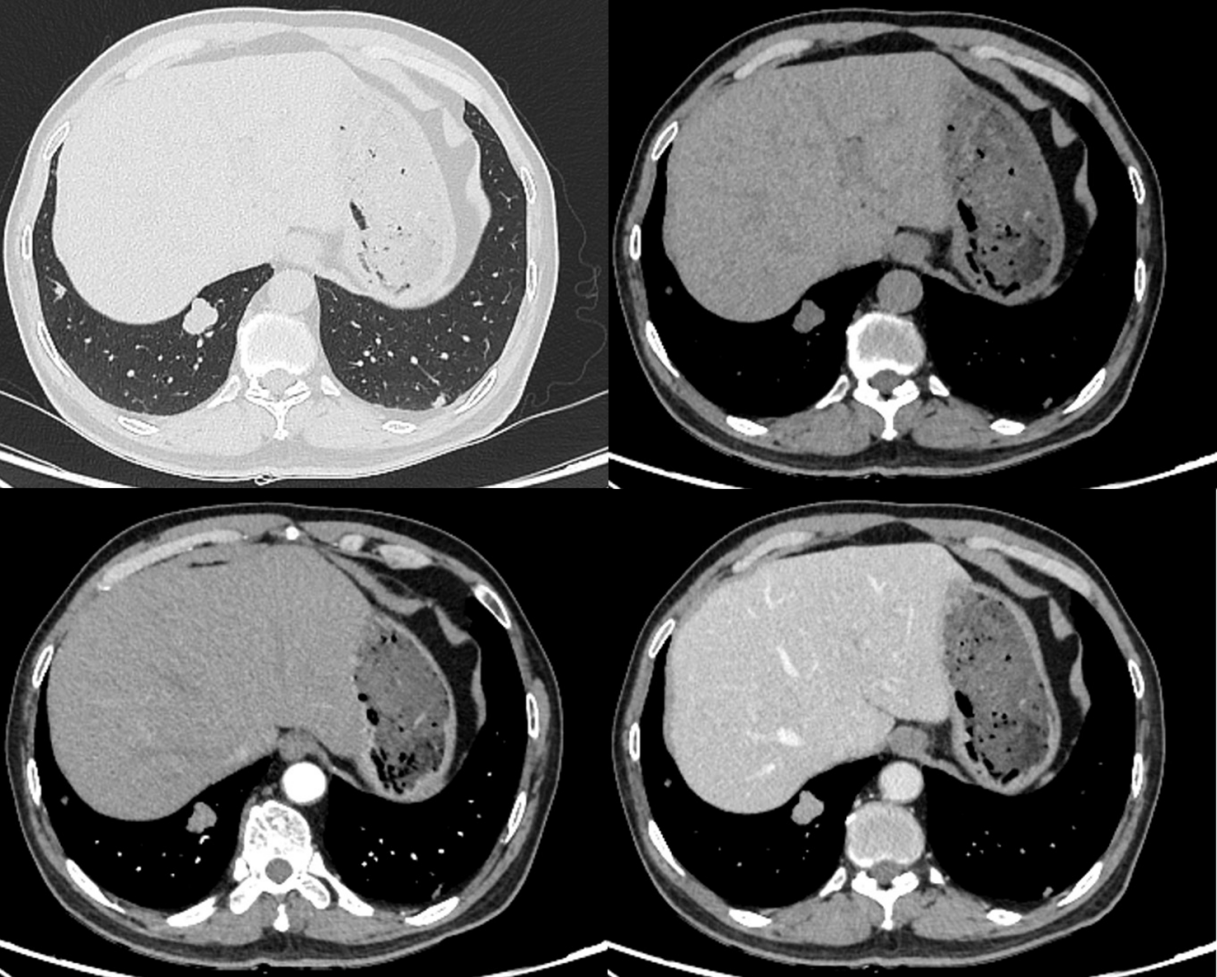
Non-contrast and contrast-enhanced CT demonstrate a well-circumscribed lobulated solid nodule (22 × 18 × 20 mm) in the right lower lobe with smooth margins, showing moderate homogeneous enhancement (attenuation values: 31.6 HU non-contrast; 69.7 HU arterial phase; 78.2 HU venous phase).

A 64-year-old male presented with progressive emaciation lasting over one year, accompanied by a recent unintentional weight loss of 4 kg. Additional symptoms included xerostomia and polydipsia. The patient denied other systemic symptoms, maintained normal mental status, regular sleep patterns, and unremarkable urinary/defecatory function. Medical history revealed type 2 diabetes mellitus managed with oral acarbose and insulin therapy. There was no history of pulmonary disease, malignancy, tobacco use, or alcohol consumption. Occupational history indicated agricultural work. Non-contrast CT demonstrated an irregular solid nodule (22 × 18 × 20 mm in transverse × anteroposterior × craniocaudal dimensions) in the posterior basal segment of the right lower lobe. The lesion exhibited lobulated contours with well-circumscribed margins and homogeneous soft-tissue attenuation (mean density ~31.6 HU), showing no internal cavitation, vacuolation, calcification or fat-density component. The periphery showed no spiculation or vascular convergence signs. On contrast-enhanced CT, the nodule exhibited arterial phase enhancement to ~69.7 HU with further increased to ~78.2 HU in the venous phase (**Figure 1**). The nodule was considered a tumor lesion, and biopsy was recommended.

**Figure 2:**
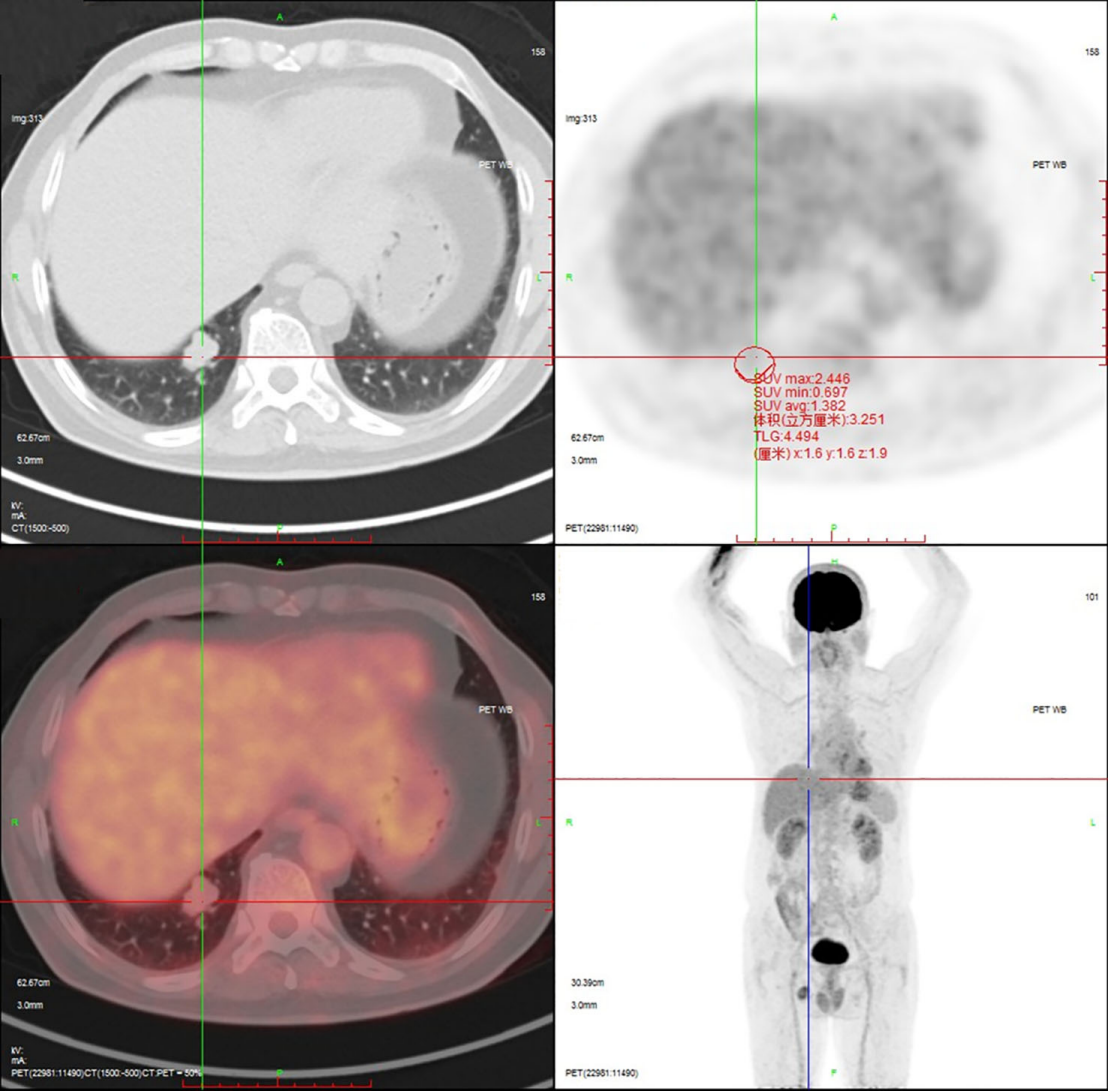
^18^F-FDG PET-CT demonstrates mild metabolic activity within the nodule, exhibiting a SUVmax of 2.4.

**Figure 3:**
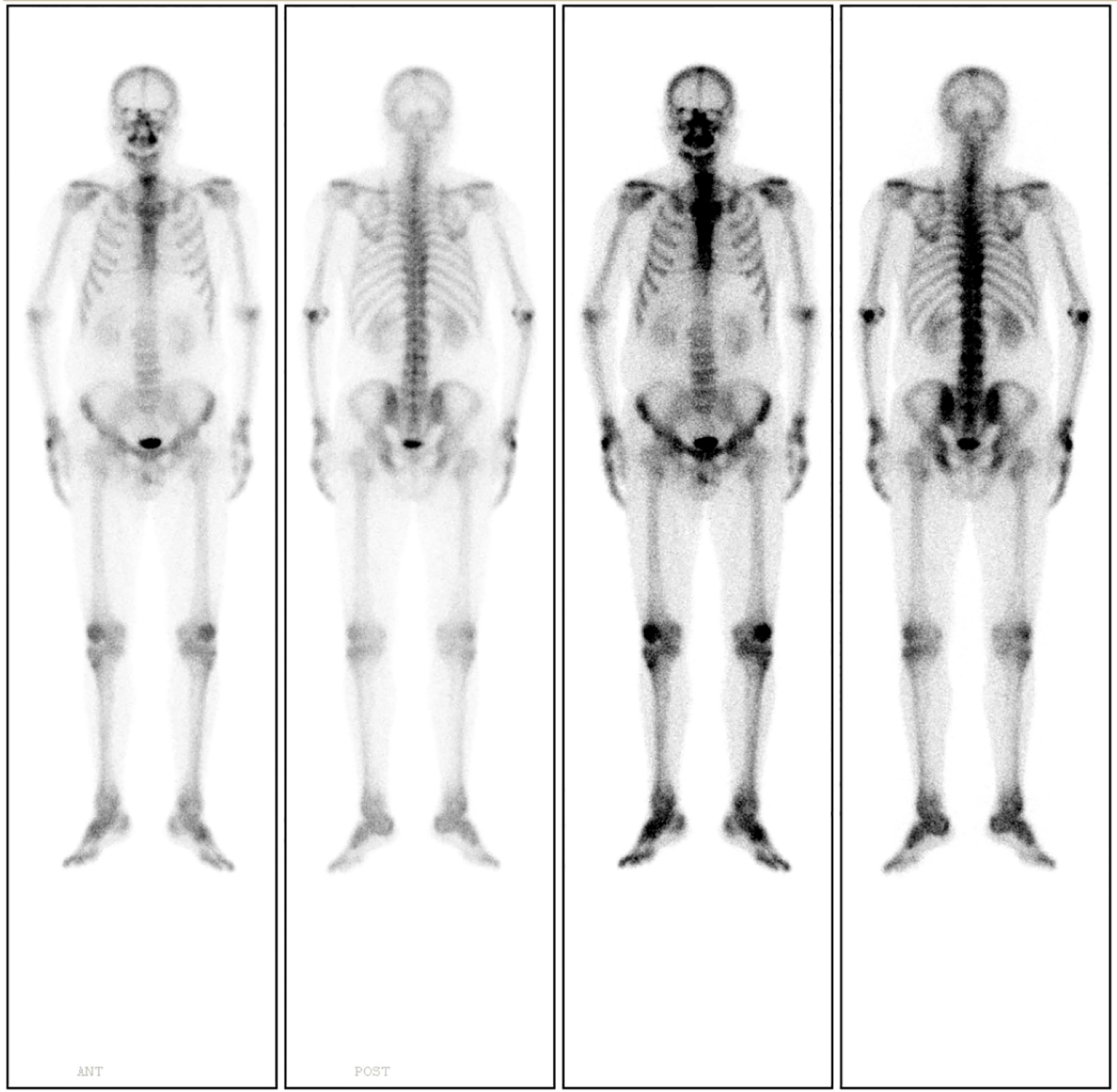
Whole-body bone scintigraphy using ECT demonstrates no evidence of pathological skeletal lesions.

18F-Fluorodeoxyglucose Positron Emission Tomography-Computed Tomography (^18^F-FDG PET-CT) imaging demonstrated mild metabolic activity within the nodule, exhibiting a maximum standardized uptake value (SUVmax) of 2.4 (**Figure 2**). The imaging findings were indeterminate between granulomatous inflammation and neoplastic lesions, necessitating histopathological confirmation via biopsy. Whole-body bone scintigraphy using emission computed tomography (ECT) revealed no evidence of pathological skeletal lesions (**Figure 3**). All laboratory parameters were within normal limits: negative for lung cancer biomarkers (CEA, NSE, CYFRA 21-1, ProGRP) and unremarkable hematological/inflammatory profiles (WBC, neutrophils, CRP, ESR).

**Figure 4:**
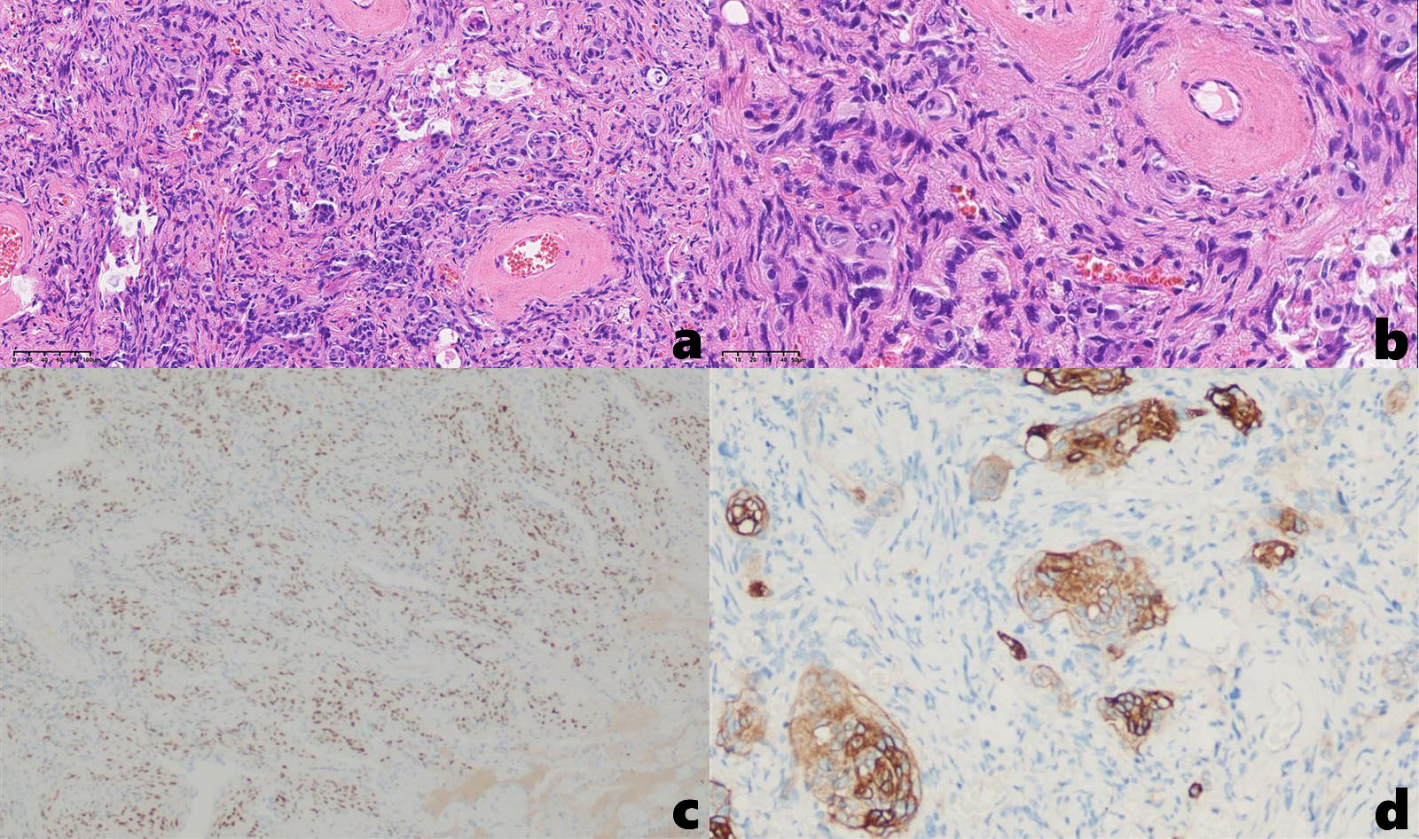
**(a, b)** H&E staining demonstrates interlacing epithelioid and spindle-shaped cells with characteristic whorled formations [**(a)**. 20×, **(b)** 40×]; Immunohistochemical results: **(c)** PR (+), **(d)** SSTR2A (local+).

Under general anesthesia, the patient underwent uniportal video-assisted thoracoscopic surgery (VATS) for wedge resection of the right lower lobe and lysis of pleural adhesions. Histopathological examination of the postoperative specimen: Hematoxylin and eosin (H&E) staining revealed interlacing arrangements of epithelioid and spindle-shaped cells with characteristic whorled formations, without evidence of nuclear atypia or abnormal mitotic figures. Immunohistochemical results: CK(-), Nestin(-), Vim(+), S-100(-), SSTR2A(local+), PR(+), EMA(+), Ki-67(approximately 1%+), PHH3(-), CD34(partially+), GFAP(-), CK7(-), TTF-1(-), Calponin(-), Desmin(-), CD31(-), Melan-A(-), HMB45(-), Bcl-2(-), SMA(-) (**Figure 4**). The morphological and immunohistochemical findings support transitional meningioma (WHO grade I). The patient recovered well postoperatively. A 12-month postoperative chest CT scan demonstrated no definitive evidence of tumor recurrence. At postoperative month 21, the patient reported no significant complaints.

## Discussion

PPM represents an exceptionally rare pulmonary neoplasm. According to the 2021 WHO classification of thoracic tumors, PPM is classified as a tumor of ectopic origin (4). PPM was first reported by Kemnitz in 1982 (5), with the majority of studies reporting benign tumors (6).

The origin of PPM is not yet clear, and some scholars have proposed hypotheses, including intrathoracic differentiation of meningeal or arachnoid cells, as well as ectopic proliferation of arachnoid cells (1, 7). PPM is typically discovered incidentally on chest CT and usually presents without symptoms (6, 8–10); nevertheless, hemoptysis may occasionally occur (11, 12). PPM often appears coin-shaped on chest X-rays and as single or multiple solid nodules on CT, with clear or lobulated boundaries and no calcification or cavities within the nodules (1, 6, 7, 10–12). In some cases, they present as pulmonary masses, ground-glass nodules, and sub-solid nodules (8, 9). ^18^F-FDG PET-CT imaging shows increased uptake of lesions (10, 13). The existing literature exhibits notable limitations in characterizing the radiological features of PPM. Comprehensive analyses of its imaging manifestations, particularly on contrast-enhanced CT and PET-CT, remain insufficiently documented. Furthermore, while pathological differentiation has been explored in some studies, the radiological criteria for differential diagnosis of PPM have received comparatively less attention in published works. Therefore, in this study, we have systematically reviewed the literature on PPM, summarized its characteristic imaging features, and comprehensively discussed the preoperative imaging-based differential diagnosis, aiming to improve clinical recognition of this condition.

On non-contrast CT, most PPMs present as well-circumscribed, round or lobulated solid nodules or masses with homogeneous density. They may be solitary or multiple and lack internal cavitation, vacuoles, calcification, or fat components. Contrast-enhanced CT demonstrates moderate enhancement of the lesions. On ^18^F-FDG PET-CT imaging, PPM exhibits increased tracer uptake. However, chest radiography was not performed in our case series, consequently, the ‘coin-shaped’ appearance was not observed. Solitary PPM requires differentiation from other primary benign pulmonary neoplasms, such as pulmonary hamartoma, pulmonary sclerosing pneumocytoma (PSP), and pulmonary inflammatory myofibroblastic tumor (IMT). Pulmonary hamartoma typically presents asymptomatically in most patients. On non-contrast CT imaging, it manifests as a solitary pulmonary nodule with well-defined, smooth margins. Intralesional fat and calcification are frequently observed, with popcorn-like calcification representing the classic presentation. The lesion demonstrates no significant enhancement on contrast-enhanced scans (14). PET-CT reveals no significant or only mild FDG uptake within the lesion (15). PSP is typically asymptomatic in the majority of patients, while a minority may present with cough, hemoptysis, or chest pain. CT imaging demonstrates a solitary, round or oval pulmonary nodule with smooth margins. The lesion is frequently isodense and may exhibit calcification. Characteristic findings include the marginal pseudocapsule sign and the air crescent sign. Following contrast administration, the lesion shows marked enhancement, and the overlying vessel sign is often observed (16, 17). On PET-CT, FDG uptake increases with lesion size, with the SUVmax potentially reaching up to 12.5 (18). IMT is frequently asymptomatic. On CT imaging, it typically presents as a solitary, round nodule located in the lung periphery, demonstrating smooth margins and heterogeneous density. Punctuate calcifications may be observed. Following contrast administration, the lesion usually exhibits heterogeneous enhancement. PET-CT demonstrates increased FDG uptake within the lesion (19). Furthermore, differential diagnoses should include other rare benign pulmonary tumors, such as benign mesenchymal tumors and benign neurogenic tumors. Additionally, solitary PPM require differentiation from tuberculoma. Typical CT findings of tuberculoma include smooth margins with an absence of or minimal lobulation, frequent calcifications, and a central low-density area representing caseous necrosis or cavitation. Satellite lesions are commonly observed surrounding the primary lesion. On contrast-enhanced CT, tuberculomas typically exhibit minimal enhancement or thin-walled rim enhancement (20). PET-CT may demonstrate significantly increased FDG uptake within tuberculomas, with the SUVmax typically ranging between 0.0 and 7.0 (21). Both solitary and multiple PPMs require differentiation from pulmonary metastases. Patients with pulmonary metastases, whether presenting as a solitary nodule or multiple nodules, invariably have a documented history of an extrathoracic primary malignancy. On CT imaging, metastases manifest as one or more nodules exhibiting varying sizes and well-defined margins. These lesions are randomly distributed throughout the lung parenchyma and typically demonstrate rapid growth on serial imaging studies.

The diagnosis of PPM requires fulfillment of two essential criteria: (1) histopathological confirmation of meningioma characteristics in the pulmonary mass or nodule, and (2) definitive exclusion of central nervous system lesions through comprehensive radiological evaluation (9). In our case, the HE staining demonstrated interlacing epithelioid and spindle-shaped cells, accompanied by whorled formations surrounding vascular structures. Immunohistochemical profiling demonstrated diffuse immunoreactivity for vimentin, progesterone receptor (PR), and epithelial membrane antigen (EMA), with focal SSTR2A positivity - a staining pattern pathognomonic for meningioma. In addition, ^18^F-FDG PET-CT imaging demonstrated no significant metabolic uptake in intracranial or extracranial regions, thereby supporting the diagnosis of PPM. Complete surgical resection remains the mainstay of treatment for PPM, with favorable postoperative outcomes achievable in most cases.

In summary, PPM is an extremely rare pulmonary neoplasm characterized predominantly by benign biological behavior. The majority of cases remain clinically asymptomatic, though hemoptysis may occasionally manifest. Radiologically, CT typically demonstrates well-circumscribed solitary or multiple solid nodules exhibiting homogeneous density with smooth margins, while characteristically lacking internal cavitation, vacuolization, calcification, or fat components. Contrast-enhanced CT reveals moderate homogeneous enhancement, while ^18^F-FDG PET-CT typically shows moderate metabolic uptake. Definitive treatment involves complete surgical resection, which achieves excellent prognosis in benign cases. PPM primarily requires differentiation from common benign pulmonary neoplasms and solitary pulmonary metastases, with definitive diagnosis dependent on pathological examination. However, our report has several limitations. Follow-up duration was relatively short (21 months), precluding assessment of long-term postoperative outcomes. Furthermore, this single-case report necessitates reliance on literature review to characterize PPM’s imaging features. Consequently, despite summarizing the imaging characteristics and detailing the differential diagnosis, PPM continues to pose diagnostic challenges that require multidisciplinary evaluation.

## Data Availability

The raw data supporting the conclusions of this article will be made available by the authors, without undue reservation.

## References

[1] Ueno M Fujiyama J Yamazaki I Uchiyama T Ishikawa Y Satoh Y . Cytology of primary pulmonary meningioma. Report of the first multiple case. Acta Cytol. (1998) 42:1424–30. doi: 10.1159/000332179, PMID: 9850654

[2] de Perrot M Kurt AM Robert J Spiliopoulos A . Primary pulmonary meningioma presenting as lung metastasis. Scand Cardiovasc J. (1999) 33:121–3. doi: 10.1080/14017439950141948, PMID: 10225315

[3] Picquet J Valo I Jousset Y Enon B . Primary pulmonary meningioma first suspected of being a lung metastasis. Ann Thorac Surg. (2005) 79:1407–9. doi: 10.1016/j.athoracsur.2003.10.071, PMID: 15797095

[4] Nicholson AG Tsao MS Beasley MB Borczuk AC Brambilla E Cooper WA . The 2021 WHO Classification of Lung Tumors: Impact of Advances Since 2015. J Thorac Oncol. (2022) 17:362–87. doi: 10.1016/j.jtho.2021.11.003, PMID: 34808341

[5] Kemnitz P Spormann H Heinrich P . Meningioma of Lung: First Report with Light and Electron Microscopic Findings. Ultrastruct Pathol. (1982) 3:359–65. doi: 10.3109/01913128209018558, PMID: 7157498

[6] Hsu CC Tsai YM Yang SF Hsu JS . Primary pulmonary meningioma. Kaohsiung J Med Sci. (2023) 39:1155–6. doi: 10.1002/kjm2.v39.11 PMC11895961 37698283

[7] Satoh Y Ishikawa Y . Primary pulmonary meningioma: Ten-year follow-up findings for a multiple case, implying a benign biological nature. J Thorac Cardiovasc Surg. (2010) 139:e39–40. doi: 10.1016/j.jtcvs.2008.07.059, PMID: 19660266

[8] Juan CM Chen ML Ho SY Huang YC . Primary Pulmonary Meningioma Simulating a Pulmonary Metastasis. Case Rep Pulmonol. (2016) 2016:8248749. doi: 10.1155/2016/8248749, PMID: 27974986 PMC5128704

[9] Chiarelli M De Simone M Gerosa M Guttadauro A Cioffi U . An incidental pulmonary meningioma revealing an intracranial meningioma: primary or secondary lesion? Ann Thorac Surg. (2015) 99:e83–84. doi: 10.1016/j.athoracsur.2015.01.045, PMID: 25841855

[10] Meirelles GS Ravizzini G Moreira AL Akhurst T . Primary pulmonary meningioma manifesting as a solitary pulmonary nodule with a false-positive PET scan. J Thorac Imaging. (2006) 21:225–7. doi: 10.1097/01.rti.0000203639.66629.68, PMID: 16915069

[11] Izumi N Nishiyama N Iwata T Nagano K Tsukioka T Hanada S and Suehiro S . Primary pulmonary meningioma presenting with hemoptysis on exertion. Ann Thorac Surg. (2009) 88:647–8. doi: 10.1016/j.athoracsur.2008.12.058, PMID: 19632430

[12] Incarbone M Ceresoli GL Di Tommaso L Cappuzzo F Inzirillo F Infante M . Primary pulmonary meningioma: report of a case and review of the literature. Lung Cancer. (2008) 62:401–7. doi: 10.1016/j.lungcan.2008.03.031, PMID: 18486986

[13] Zhang DB Chen T . Primary pulmonary meningioma: A case report and review of the literature. World J Clin cases. (2022) 10:4196–206. doi: 10.12998/wjcc.v10.i13.4196, PMID: 35665099 PMC9131207

[14] Sodhi KS Virmani V Jindal SK Khandelwal N . Pulmonary hamartoma. Ann Acad Med Singap. (2009) 38:1110. doi: 10.47102/annals-acadmedsg, PMID: 20052452

[15] Ye S Meng S Bian S Zhao C Yang J Lei W . Diagnosis value of (18)F-Fluoro-D-glucose positron emission tomography-computed tomography in pulmonary hamartoma: a retrospective study and systematic review. BMC Med Imaging. (2023) 23:28. doi: 10.1186/s12880-023-00981-z, PMID: 36747135 PMC9903478

[16] Shin SY Kim MY Oh SY Lee HJ Hong SA Jang SJ . Pulmonary sclerosing pneumocytoma of the lung: CT characteristics in a large series of a tertiary referral center. Med (Baltimore). (2015) 94:e498. doi: 10.1097/MD.0000000000000498, PMID: 25634202 PMC4602969

[17] Wang QB Chen YQ Shen JJ Zhang C Song B Zhu XJ . Sixteen cases of pulmonary sclerosing haemangioma: CT findings are not definitive for preoperative diagnosis. Clin Radiol. (2011) 66:708–14. doi: 10.1016/j.crad.2011.03.002, PMID: 21529795

[18] Xu J Dong Y Yin G Jiang W Yang Z Xu W . (18) F-FDG PET/CT imaging: A supplementary understanding of pulmonary sclerosing pneumocytoma. Thorac Cancer. (2019) 10:1552–60. doi: 10.1111/tca.2019.10.issue-7, PMID: 31131992 PMC6610286

[19] Khatri A Agrawal A Sikachi RR Mehta D Sahni S Meena N . Inflammatory myofibroblastic tumor of the lung. Adv Respir Med. (2018) 86:27–35. doi: 10.5603/ARM.2018.0007, PMID: 29490419

[20] Totanarungroj K Chaopotong S Tongdee T . Distinguishing small primary lung cancer from pulmonary tuberculoma using 64-slices multidetector CT. J Med Assoc Thai. (2012) 95:574–82., PMID: 22612014

[21] Goo JM Im JG Do KH Yeo JS Seo JB Kim HY . Pulmonary tuberculoma evaluated by means of FDG PET: findings in 10 cases. Radiology. (2000) 216:117–21. doi: 10.1148/radiology.216.1.r00jl19117, PMID: 10887236

